# Identifying Ectopic Pregnancy in a Large Integrated Health Care Delivery System: Algorithm Validation

**DOI:** 10.2196/18559

**Published:** 2020-11-30

**Authors:** Darios Getahun, Jiaxiao M Shi, Malini Chandra, Michael J Fassett, Stacey Alexeeff, Theresa M Im, Vicki Y Chiu, Mary Anne Armstrong, Fagen Xie, Julie Stern, Harpreet S Takhar, Alex Asiimwe, Tina Raine-Bennett

**Affiliations:** 1 Department of Research & Evaluation Kaiser Permanente Southern California Pasadena, CA United States; 2 Department of Health Systems Science Kaiser Permanente Bernard J. Tyson School of Medicine Pasadena, CA United States; 3 Division of Research Kaiser Permanente Northern California Oakland, CA United States; 4 Department of Obstetrics and Gynecology Kaiser Permanente West Los Angeles Medical Center Los Angeles, CA United States; 5 Bayer AG Berlin Germany

**Keywords:** ectopic pregnancy, pregnancy, validation, predictive value, electronic health records, electronic database

## Abstract

**Background:**

Surveillance of ectopic pregnancy (EP) using electronic databases is important. To our knowledge, no published study has assessed the validity of EP case ascertainment using electronic health records.

**Objective:**

We aimed to assess the validity of an enhanced version of a previously validated algorithm, which used a combination of encounters with EP-related diagnostic/procedure codes and methotrexate injections.

**Methods:**

Medical records of 500 women aged 15-44 years with membership at Kaiser Permanente Southern and Northern California between 2009 and 2018 and a potential EP were randomly selected for chart review, and true cases were identified. The enhanced algorithm included diagnostic/procedure codes from the International Classification of Diseases, Tenth Revision, used telephone appointment visits, and excluded cases with only abdominal EP diagnosis codes. The sensitivity, specificity, positive predictive value (PPV), negative predictive value (NPV), and overall performance (Youden index and F-score) of the algorithm were evaluated and compared to the validated algorithm.

**Results:**

There were 334 true positive and 166 true negative EP cases with available records. True positive and true negative EP cases did not differ significantly according to maternal age, race/ethnicity, and smoking status. EP cases with only one encounter and non-tubal EPs were more likely to be misclassified. The sensitivity, specificity, PPV, and NPV of the enhanced algorithm for EP were 97.6%, 84.9%, 92.9%, and 94.6%, respectively. The Youden index and F-score were 82.5% and 95.2%, respectively. The sensitivity and NPV were lower for the previously published algorithm at 94.3% and 88.1%, respectively. The sensitivity of surgical procedure codes from electronic chart abstraction to correctly identify surgical management was 91.9%. The overall accuracy, defined as the percentage of EP cases with correct management (surgical, medical, and unclassified) identified by electronic chart abstraction, was 92.3%.

**Conclusions:**

The performance of the enhanced algorithm for EP case ascertainment in integrated health care databases is adequate to allow for use in future epidemiological studies. Use of this algorithm will likely result in better capture of true EP cases than the previously validated algorithm.

## Introduction

Use of claims, administrative databases, and electronic health records (EHRs) allows for efficient identification of individuals with medical conditions [1]. National hospital databases and discharge diagnoses have been used extensively to monitor serious medical conditions leading to significant morbidity such as acute myocardial infarction; however, hospital databases are not sufficient in capturing serious conditions that do not necessarily require hospitalization. Ectopic pregnancy (EP), the implantation of a fertilized ovum outside of the endometrial cavity, is a serious condition that can be life threatening; however, a significant proportion of patients can be managed in the outpatient setting. Trends in EP are difficult to examine because women with EPs are increasingly managed in the outpatient setting, either medically with methotrexate injection(s) or surgically with laparoscopy [2,3]. Furthermore, women with potential EPs may be evaluated over the course of several days and medical encounters prior to the establishment of a definitive diagnosis of EP or viable or nonviable intrauterine pregnancy, making identification of true cases difficult.

Researchers have typically relied on clinical diagnosis and procedure codes extracted from outpatient care and hospital discharge databases to describe trends in EP. However, the accuracy of EP case ascertainment and the validity of study findings depend on the types of data sources and completeness of EP case ascertainment approaches. One methodology for EP case ascertainment was validated in a study by Scholes et al in 2011 [4], using claims and administrative data extracted from a large health care maintenance organization database prior to the use of EHRs and codes from the International Classification of Diseases, Tenth Revision (ICD-10). Although the sensitivity of the algorithm for capturing EP cases was higher than that of the use of standard codes, the algorithm is inherently limited by the time frame of the study, the completeness of the data, and the ability to review patients' medical information in an electronic database for true case ascertainment [5-8].

The widespread adoption of EHRs in the United States presents an opportunity to improve patient care [9,10] and provides researchers unparalleled possibilities to conduct high-quality clinical and pharmacoepidemiologic research [11,12]. EHRs provide access to more reliable and comprehensive patient health information. They are also easily transferable to other EHR systems and more cost-efficient than paper-based data sources [13-15]. Over the last decade, there have been a number of studies that evaluated the accuracy of health data (hospital discharge data, outpatient encounter data, and claims data) extracted from the EHRs of various regions of the Kaiser Permanente health care system [16-19] and other health care systems [20,21]. Published validation studies investigated demographic characteristics [17], body weight and height data [22], perinatal outcomes [18,23], phenotype for genomic study [21], and phenotype of HIV infection [20]. However, to our knowledge, there is no study that has assessed the validity of EP case ascertainment using EHRs for validation and the potential impact of changes in the data over time (pre-EHR vs EHR era). There is substantial practice pattern variation over time, across institutions and health care providers. The Scholes et al algorithm was developed 10 years ago at two institutions with potentially different practice environments than the setting of this study. Furthermore, the data for the Scholes et al algorithm came largely from contracting hospitals for inpatient care, which may have disparate practice and coding patterns. Therefore, validating the algorithm in a different time frame and setting is necessary to conduct future studies describing the temporal trends of EP incidence and treatment modalities. This study aimed to develop an enhanced algorithm that builds on the previously validated algorithm [4].

## Methods

Kaiser Permanente Northern California (KPNC) and Southern California (KPSC) are the two largest Kaiser Permanente regions of the nine regional entities in the United States. These integrated health care systems provide health care service to over 9 million racially and ethnically diverse members who receive their care mainly from KP physicians and allied staff in 36 hospitals and over 427 medical centers scattered throughout California. Both KPSC and KPNC access the Virtual Data Warehouse, which was created to facilitate multi-site research projects. KP health care staff in both outpatient and inpatient clinical settings utilize an EHR based on an Epic platform that is accessible to multiple health care providers at the same time and in multiple locations. KPSC and KPNC fully implemented the EHR system for both outpatient care encounters and inpatient services in 2008 and 2009, respectively. It is a highly sophisticated integrated health information management and care management system designed to enhance the quality of patient care. The data is collected in real time with patient-centered records that provide access to comprehensive patient information to clinicians and researchers more instantly, efficiently, and securely compared with pre-EHR era paper records.

We developed an enhanced algorithm to identify EPs in the two health care systems through several iterative steps: First, we incorporated corresponding ICD-10 diagnostic and procedure codes that were not in use when the Scholes et al algorithm was developed in 2011. We then chart reviewed an initial random sample of 100 cases (50 KPNC and 50 KPSC) that had at least one EP diagnostic or procedure code but were not classified as EP by the Scholes et al algorithm to understand the reasons for misclassification. This information was used to modify the Scholes et al algorithm to improve the accuracy of case ascertainment. In addition to the inclusion of ICD-10 diagnostic/procedure codes, the major changes that were made to the previously validated algorithm as a result of our initial chart review were the addition of a new source of information (telephone appointment visits [TAVs]), the exclusion of cases with only abdominal EP diagnosis codes, additional criteria of a combination of an EP diagnostic and procedure code to be considered a case, refinement of methotrexate medication codes that were considered valid, and expansion of the allowable days from the assigned EP diagnosis date to administration of methotrexate.

**Figure 1:**
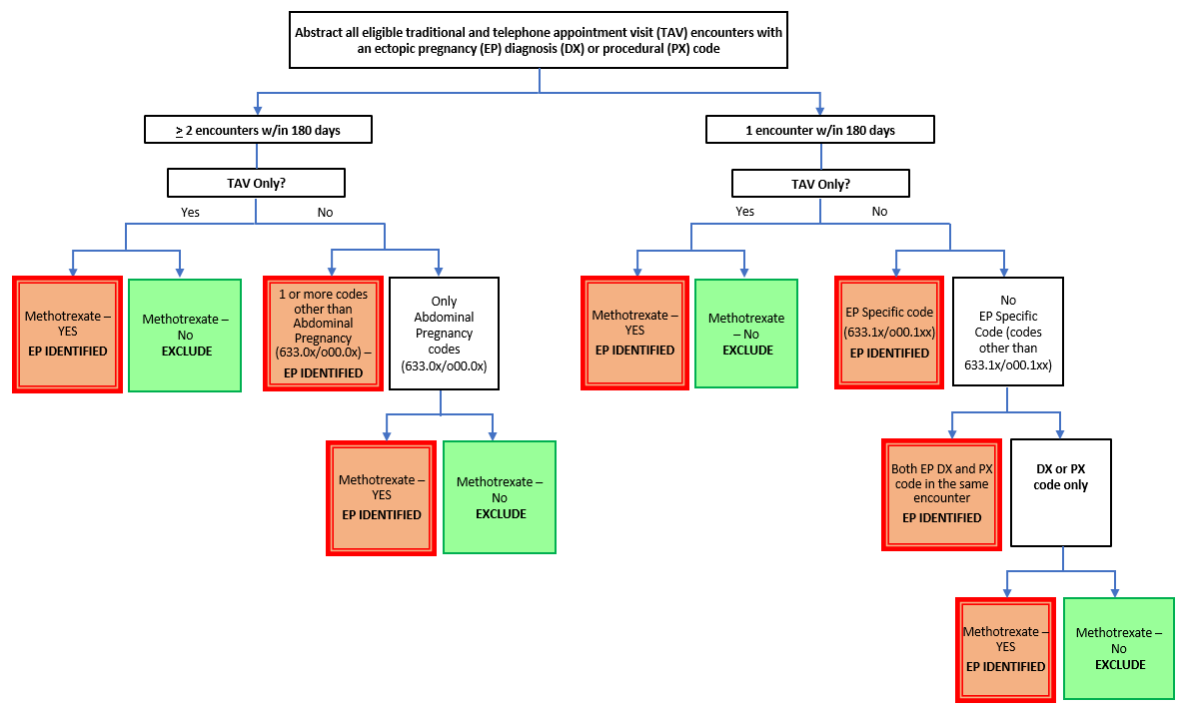
Enhanced ectopic pregnancy algorithm.

The final enhanced algorithm (Figure 1) that was developed required either (1) at least 2 encounters, including at least 1 in-person visit, with an EP code other than abdominal EP (abdominal codes O00.00 and O00.01); (2) at least 2 TAVs with an EP code and evidence of methotrexate use; (3) at least 1 outpatient or inpatient visit or outside claims visit with any of the specific ICD, Ninth Revision (ICD-9), or ICD-10 diagnostic codes 633.10, 633.11, O00.10, and O00.11; (4) a combination of any single encounter (outpatient or inpatient visit, outside claims visit, or TAV) with a nonspecific EP code plus evidence of methotrexate use; or (5) a single non-TAV encounter with both an EP diagnosis and procedure code on the same encounter.

The EP diagnosis date was defined as the date of the first encounter with an EP code. Multiple encounters with EP codes occurring within a 180-day period from the first encounter with an EP code were considered part of the same pregnancy episode. Methotrexate use was defined as a medication code found within 30 days prior to and 180 days after the first EP diagnosis date. The justification for relaxing the criteria for methotrexate administration to 30 days prior to the first diagnosis, in contrast to the 7 days allowed in Scholes et al algorithm, was to minimize misclassification of treatment status due to inaccurate assignment of EP diagnosis dates. In randomly selected chart abstractions, we also found that methotrexate medication codes had various administrative subcodes that corresponded with true use of methotrexate; hence, we had to specify medication administration subcodes.

**Figure 2:**
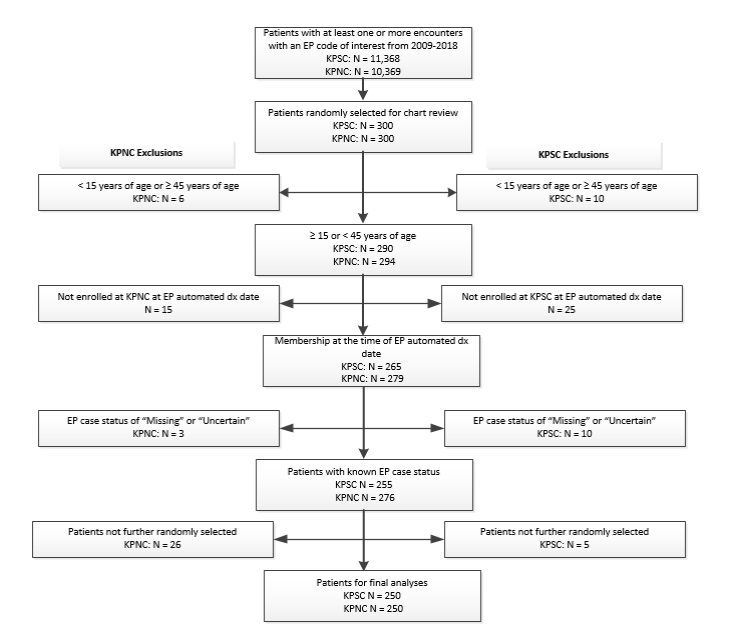
Flow diagram of validation study sample. EP: ectopic pregnancy. KPNC: Kaiser Permanente Northern California. KPSC: Kaiser Permanente Southern California.

To assess the validity of the previously validated algorithm by Scholes et al and the newly developed enhanced version of the algorithm against the gold-standard “true case” as determined by chart review, a random sample of 600 patients (300 at each site) with a potential EP was selected. A potential case was defined as any case with at least 1 ICD-9, ICD-10, or Current Procedural Terminology code for EP (Multimedia Appendix 1). This approach was chosen because, in our setting, as in most health care settings that rely on insurance reimbursement, it is unlikely for an EP case to not have documentation with either a diagnosis or procedural code. Therefore, we assumed that cases that did not meet the initial inclusion criteria would be very unlikely to be a true EP case. By limiting the sample to cases with these inclusion criteria, we increased the number of true cases with little risk of missing cases. Further inclusion criteria were applied (women who were aged 15 to 44 years from January 1, 2009, to December 31, 2018, and were enrolled in the health plan for at least 1 month over the study period) to the 600 randomly selected cases. Cases that did not meet these requirements were excluded, leaving 255 cases at KPSC and 276 at KPNC. We randomly selected 250 cases from each site for chart review for this validation study (Figure 2).

Using a standardized abstraction form, chart reviews were performed by trained abstractors to identify true EP cases. Cases where EP status was unclear were identified and adjudicated by a clinician. In our analysis of preliminary data pulls, we found that 10.5% (1568/14,907) of EP cases identified using the Scholes et al algorithm for classification could not be clearly classified as either medical or surgical. Therefore, information on treatment modality (surgical vs medical) was collected to assess the level of agreement. EP cases were classified as surgically managed if the patient had undergone any EP removal surgery within 30 days of the first encounter with an EP code, regardless of whether the patient received methotrexate. Remaining EP cases were classified as medically treated if the patient received methotrexate for an EP. Cases for which the type of treatment could not be determined were considered unclassified.

The test performance of both algorithms was calculated on the 500 potential EP cases: sensitivity (percentage of chart review–confirmed cases that were correctly classified as EP by the algorithm), specificity (percentage of cases determined not to be EP by chart review that were correctly classified by the algorithm), positive predictive value (PPV; percentage of cases classified as EP by the algorithm that were confirmed by chart review), and negative predictive value (NPV; percentage of identified cases classified as not EP by the algorithm that were determined not to be EP cases from chart review). Furthermore, the overall test performance of a dichotomous diagnostic test was assessed using the Youden J statistic [24] (Youden index=sensitivity+specificity–1), and the weighted harmonic mean of the test's precision and recall were assessed by computing the F-score (2×[PPV×sensitivity]/[PPV+sensitivity]). Agreement in case identification between the Scholes et al and the enhanced algorithms was assessed using kappa (κ) statistics. In addition, we evaluated the performance of electronic abstraction in correctly identifying EP management type (medical or surgical) among confirmed EP cases compared to that of chart review using the same performance measures. Lastly, we conducted a sensitivity analysis calculating the same performance measures using the Scholes et al algorithm and enhanced algorithm for a subset of cases from 2009 to the end of 2014 (ICD-9–only cases).

## Results

Table 1 shows the distribution of maternal characteristics among the study sample and the two study sites (KPSC and KPNC) from which the sample for this validation study was drawn. Only a small proportion of the women in the sample population were teens and over a third were Hispanic. There was a higher proportion of Hispanic members at KPSC than at KPNC and a higher proportion of non-Hispanic White and Asian/Pacific Islander members at KPNC than at KPSC. Only a small proportion of women in the sampled cohort lived in neighborhoods with a median annual household income below US $30,000. Although the distribution of maternal characteristics is largely comparable between the sampled population and the overall cohort, women in the sampled population were slightly more likely to be from non-Hispanic Black backgrounds and less likely to be from non-Hispanic White racial/ethnic backgrounds.

**Table 1:** Characteristics of the validation study sample and the combined and site-specific populations.

Characteristics		Sample (n=500)	KPSC^a^ and KPNC^b^ populations		
		Chart reviewed, n (%)	Overall, n (%) (N=19,615)	KPSC, n (%) (n=9823)	KPNC, n (%) (n=9792)
**Maternal age (years)**					
	<20	22 (4.4)	668 (3.4)	353 (3.6)	315 (3.2)
	20-29	169 (33.8)	7036 (35.9)	3643 (37.1)	3393 (34.7)
	30-34	157 (31.4)	6073 (31.0)	2970 (30.2)	3103 (31.7)
	≥35	152 (30.4)	5838 (29.8)	2857 (29.1)	2981 (30.4)
**Race/ethnicity**					
	Non-Hispanic White	124 (24.8)	5458 (27.8)	2257 (23.0)	3201 (32.7)
	Non-Hispanic Black	82 (16.4)	2579 (13.1)	1298 (13.2)	1281 (13.1)
	Hispanic	199 (39.8)	7668 (39.1)	4960 (50.5)	2708 (27.7)
	Asian/Pacific Islander	83 (16.6)	3261 (16.6)	1069 (10.9)	2192 (22.4)
	Other	4 (0.8)	349 (1.8)	144 (1.5)	205 (2.1)
	Unknown	8 (1.6)	300 (1.5)	95 (1.0)	205 (2.1)
**Smoking status^c^**					
	No	461 (92.2)	17,947 (91.5)	8929 (90.9)	9018 (92.1)
	Yes	39 (7.8)	1668 (8.5)	894 (9.1)	774 (7.9)
**Parity**					
	Nullipara	146 (29.2)	5690 (29.0)	2671 (27.2)	3019 (30.8)
	Multipara	259 (51.8)	10,444 (53.2)	5214 (53.1)	5230 (53.4)
	Missing/unavailable	95 (19.0)	3481 (17.7)	1938 (19.7)	1543 (15.8)
**Family household income^d^ (US $)**					
	<$30,000	31 (6.2)	1092 (5.6)	584 (5.9)	508 (5.2)
	$30,000-$49,999	117 (23.4)	4863 (24.8)	2806 (28.6)	2057 (21.0)
	$50,000-$69,999	147 (29.4)	5474 (27.9)	2913 (29.7)	2561 (26.2)
	$70,000-$89,999	104 (20.8)	4131 (21.1)	1969 (20.0)	2162 (22.1)
	≥$90,000	101 (20.2)	4033 (20.6)	1535 (15.6)	2498 (25.5)

^a^KPSC: Kaiser Permanente Southern California.

^b^KPNC: Kaiser Permanente Northern California.

^c^Smoking status documented within the year prior to the index date.

^d^Median family household income based on census tract of residence.

Chart review demonstrated that 334 (66.8%) of the 500 cases were true ectopic pregnancies. The sensitivity, specificity, PPV, and NPV of using the Scholes et al algorithm and the enhanced algorithm for identifying EPs are presented in Table 2. The sensitivity, specificity, NPV, and PPV for the Scholes et al algorithm were lower at 94.3% (315/334), 84.3% (140/166), 88.1% (140/159), and 92.4% (315/341), respectively, compared to those for the enhanced algorithm at 97.6% (326/334), 84.9% (141/166), 94.6% (141/149), and 92.9% (326/351), respectively. Furthermore, the overall performance (Youden index and F-score) of the enhanced algorithm was higher than the performance of the Scholes et al algorithm at 82.5 and 95.2 versus 78.7 and 93.3, respectively.

**Table 2:** Ectopic pregnancy ascertainment performance of the Scholes et al and enhanced ectopic pregnancy algorithms.

Characteristic			Scholes et al algorithm					Enhanced algorithm				
			Yes		No		Total		Yes		No	Total
**Classification by chart review, n**												
	Yes	315		19		334		326		8		334
	No	26		140		166		25		141		166
	Total	341		159		500		351		149		500
**Test characteristics**												
	Sensitivity, % (n/N)	N/A^a^		N/A		94.3 (315/334)		N/A		N/A		97.6 (326/334)
	Specificity, % (n/N)	N/A		N/A		84.3 (140/166)		N/A		N/A		84.9 (141/166)
	Negative predictive value, % (n/N)	N/A		N/A		88.1 (140/159)		N/A		N/A		94.6 (141/149)
	Positive predictive value, % (n/N)	N/A		N/A		92.4 (315/341)		N/A		N/A		92.9 (326/351)
	Youden index	N/A		N/A		78.6		N/A		N/A		82.5
	F-score	N/A		N/A		93.3		N/A		N/A		95.2

^a^N/A: not applicable.

We evaluated the performance of electronic abstraction in correctly identifying EP management type in the 326 EP cases identified by both the chart review and the enhanced algorithm. Chart review revealed that 197 (60.4%) were managed surgically, 126 (38.7%) were managed medically, and 3 (0.9%) could not be classified. Electronic abstraction assigned 186 (57.1%) EP cases as managed surgically and 124 (38.0%) as managed medically, and 16 (4.9%) could not be classified. The performance of electronic chart abstraction in assigning EP management compared to that of chart review is provided in Table 3. The sensitivity of surgical procedure codes from electronic chart abstraction to correctly identify surgical management was 91.9% (181/197). The overall accuracy, defined as the percentage of EP cases with correct management (surgical, medical, and unclassified) identified by electronic chart abstraction, was 92.3% (301/326). An excellent level of agreement in EP case identification (κ=0.93, 95% CI 0.89-0.96) was observed between the Scholes et al algorithm and the enhanced algorithm.

**Table 3:** Ectopic pregnancy management ascertainment performance of electronic data abstraction.

Characteristic		Classification by electronic abstraction^a^			
		Surgical	Medical	Unclassified	Total
**Classification by chart review, n**					
	Surgical	181	5	11	197
	Medical	5	118	3	126
	Unclassified	0	1	2	3
	Total	186	124	16	326^a^
**Test characteristics**					
	Sensitivity, % (n/N)	91.9 (181/197)	N/A^b^	N/A	N/A
	Specificity, % (n/N)	96.1 (124/129)	N/A	N/A	N/A
	Negative predictive value, % (n/N)	88.6 (124/140)	N/A	N/A	N/A
	Positive predictive value, % (n/N)	97.3 (181/186)	N/A	N/A	N/A
	Youden index	88	N/A	N/A	N/A
	F-score	94.5	N/A	N/A	N/A
	Overall accuracy^c^, % (n/N)	N/A	N/A	N/A	92.3 (301/326)

^a^Includes cases confirmed as ectopic pregnancy by chart review and the enhanced algorithm.

^b^N/A: not applicable.

^c^The percentage of ectopic pregnancy cases with correct management (surgical, medical, and unclassified) identified by electronic chart abstraction.

Sensitivity analysis limiting data to the subset of cases (n=307) from 2009 to 2014 with ICD-9–only codes revealed that the sensitivity and NPV for the Scholes et al subset analysis, at 94.5% (206/218) and 85.9% (73/85), respectively (Table 4), were similar to 94.3% (315/334) and 88.1% (140/159), respectively, for the Scholes et al full data set (Table 2). The performance of the enhanced algorithm in the subset analyses (sensitivity of 97.2%, 212/218; NPV of 92.4%, 73/79) was also similar to the performance of the enhanced algorithm for the full data set (sensitivity of 97.6%, 326/334; NPV of 94.6%, 141/149).

**Table 4:** Sensitivity analysis of ectopic pregnancy ascertainment performance of the Scholes et al [4] and enhanced ectopic pregnancy algorithms on a 2009-2014 ICD-9–only subset.

Characteristic		Scholes et al algorithm			Enhanced algorithm			
		Yes	No	Total		Yes	No	Total
**Classification by chart review, n**								
	Yes	206	12	218		212	6	218
	No	16	73	89		16	73	89
	Total	222	85	307		228	79	307
**Test characteristics**								
	Sensitivity, % (n/N)	N/A^a^	N/A	94.5 (206/218)		N/A	N/A	97.2 (212/218)
	Specificity, % (n/N)	N/A	N/A	82.0 (73/89)		N/A	N/A	82.0 (73/89)
	Negative predictive value, % (n/N)	N/A	N/A	85.9 (73/85)		N/A	N/A	92.4 (73/79)
	Positive predictive value, % (n/N)	N/A	N/A	92.8 (206/222)		N/A	N/A	93.0 (212/228)
	Youden index	N/A	N/A	76.5		N/A	N/A	79.3
	F-score	N/A	N/A	93.6		N/A	N/A	95.1

^a^N/A: not applicable.

## Discussion

In this validation study of EP, we found that our enhanced version of an algorithm that was previously validated by Scholes et al [4] in 2011 for identification of EP had a slightly higher sensitivity of 97.6% and negative predictive value of 94.6% compared to the original algorithm. The overall test performance, as estimated by the Youden index and F-score, was also much higher for the enhanced algorithm. However, we found similar specificities and PPVs in both the enhanced and Scholes et al algorithms. Furthermore, limiting the test performance to the pre-EHR era, the period when ICD-9 was used to code and classify medical conditions (2009-2014), the enhanced algorithm yielded a higher sensitivity, NPV, and overall test performance in EP case identification, suggesting that differences are due to improvement in clinical information collection and retrieval rather than any ICD code changes (from ICD-9 to ICD-10).

The quality of data extracted from outpatient encounters and hospital discharge records has been well studied. The accuracy of data abstraction varies by health care system, coding and clinical practice, and design of EHR query modules, among others [25]. For example, in a fee-for-service setting, in-person visits may be the primary mode of care; however, in a capitated care model, telephone encounters, which are not billable but allow providers to speak directly with patients who may be at home or another convenient location, may be used more frequently. These appointments usually last about 20 minutes and do not require a copay. Although an efficient option that helps patients avoid unnecessary in-person doctor visits, the usefulness and quality of data extracted from TAVs has not been well studied. We evaluated the performance of our enhanced algorithm after including TAV in the algorithm and found that accuracy improved when TAV EP codes were used in combination with EP codes from in-person encounters (Multimedia Appendix 2).

Scholes et al developed the original algorithm using a classification and regression tree (CART) [26]. The CART model is a nonparametric classification technique for building decision trees in which results are presented in a useful and easy-to-interpret “tree” format. However, it does not generate prediction probabilities needed to assess calibration. Model discriminatory accuracy is typically assessed. We made minor modifications to the algorithm to incorporate equivalent ICD-10 diagnostic and procedure codes and took into account other coding differences unique to the current EHRs (ie, new medication codes) and clinical practice (ie, increasing use of TAVs). Therefore, our enhanced algorithm is updated to a more current health care setting, has high PPV for case identification, and will support contemporary observational studies with validated accuracy. Since our enhanced algorithm had a higher PPV than the Scholes et al algorithm and the agreement with the Scholes algorithm was high, we did not perform a new CART analysis.

Accurate case identification using the enhanced algorithm is feasible and increasingly useful for public health disease surveillance and epidemiological studies. Furthermore, early identification of high-risk women may provide better opportunities for early detection of EP in affected women.

The overall accuracy of electronic data abstraction to identify surgical management of EP was 92.3%. Although we demonstrated a high overall accuracy using surgical codes (Table 3), consideration should also be given to using additional surgical codes for tubal surgery that were not included in the case-finding algorithm because they were not EP-related codes but may be used by some providers at the time of EP surgery in order to increase the accuracy of management assignment.

This study has strengths and limitations. The socioeconomically diverse patient population at KPNC and KPSC, which is broadly representative of California, makes our findings widely generalizable to health systems with similar clinical patterns (ie, closed health care systems). However, future research is needed to examine whether the enhanced algorithm can be applied in other settings. The validation of the enhanced algorithm based on EHRs during the time periods both prior to and subsequent to EHR implementation further enhances the strength of this study. While we attempted to identify all potential EP cases by using cases with either an EP-related diagnostic or procedure code, it is possible that EP cases that were incorrectly or not coded were not captured, which would have falsely increased the sensitivity of both algorithms. We did not adjust for the influence of baseline characteristics. Therefore, some caution in interpreting the findings is warranted.

The enhanced algorithm yielded better overall EP case identification test results from EHR data, with slight improvements in sensitivity, specificity, and predictive values compared to the algorithm developed using pre-EHR era data, suggesting that the accuracy of EP case identification can be improved by supplementing the Scholes et al algorithm with TAV and ICD-10 diagnosis and procedure codes from EHRs. Overall, the enhanced algorithm for EP case identification in integrated health care databases is adequate to allow for its use in future epidemiological studies. Further studies on the quality of EHRs geared toward specific prenatal outcomes are urgently needed.

## Appendix

Multimedia Appendix 1 International Classification of Diseases diagnostic and procedure codes (ICD-9 and ICD-10), Current Procedural Terminology (CPT-4) codes for ectopic pregnancy in the enhanced algorithm*.

Multimedia Appendix 2 Ectopic pregnancy ascertainment in telephone appointment visits - performance of electronic data abstraction.

## Abbreviations

CART: classification and regression tree
EHRs: electronic health records
EP: ectopic pregnancy
ICD-9: International Classification of Diseases, Ninth Revision
ICD-10: International Classification of Diseases, Tenth Revision
KPNC: Kaiser Permanente Northern California
KPSC: Kaiser Permanente Southern California
NIH/NICHD: National Institutes of Health/National Institute of Child Health and Human Development
NPV: negative predictive value
PPV: positive predictive value
TAVs: telephone appointment visits
