# Supplementary material for: Identifying Ectopic Pregnancy in a Large Integrated Health Care Delivery System: Algorithm Validation
Source: JMIR Med Inform. 2020 Nov 30;8(11):e18559. doi: 10.2196/18559 (PMC7735905; doi:10.2196/18559)
Supplement: Multimedia Appendix 1 [file medinform_v8i11e18559_app1.docx]

| **ICD-9 Codes** | **ICD-10 Codes** | | **CPT4 Codes** |
| --- | --- | --- | --- |
| ***Diagnostic*** | ***Diagnostic*** | | ***Surgical*** |
| 633 | O00 | O00.20 | 59120 |
| 633.00 | O00.00 | O00.201 | 59121 |
| 633.01 | O00.01 | O00.202 | 59130 |
| 633.10* | O00.1* | O00.209 | 59135 |
| 633.11* | O00.10* | O00.21 | 59136 |
| 633.20 | O00.101* | O00.211 | 59140 |
| 633.21 | O00.102* | O00.212 | 59150 |
| 633.80 | O00.109* | O00.219 | 59151 |
| 633.81 | O00.11* | O00.8 |  |
| 633.90 | O00.111* | O00.80 |  |
| 633.91 | O00.112* | O00.81 |  |
|  | O00.119* | O00.9 |  |
|  |  | O00.90 |  |
|  |  | O00.91 |  |
|  |  |  |  |
| ***Procedure*** | ***Procedure*** | |  |
| 66.62 | 10T20ZZ | 0UB58ZZ |  |
| 74.3 | 10T23ZZ | 0UB60ZZ |  |
| 66.01 | 10T24ZZ | 0UB63ZZ |  |
| 66.02 | 10T27ZZ | 0UB64ZZ |  |
|  | 10T28ZZ | 0UB67ZZ |  |
|  | 10D27ZZ | 0UB68ZZ |  |
|  | 10D28ZZ | 0UT50ZZ |  |
|  | 0UB50ZZ | 0UT54ZZ |  |
|  | 0UB53ZZ | 0UT60ZZ |  |
|  | 0UB54ZZ | 0UT64ZZ |  |
|  | 0UB57ZZ |  |  |

*Codes considered specific for ectopic pregnancy; in-person visits with ectopic pregnancy- specific codes were categorized as a case.
