# Supplementary material for: Identifying Ectopic Pregnancy in a Large Integrated Health Care Delivery System: Algorithm Validation
Source: JMIR Med Inform. 2020 Nov 30;8(11):e18559. doi: 10.2196/18559 (PMC7735905; doi:10.2196/18559)
Supplement: Multimedia Appendix 2 [file medinform_v8i11e18559_app2.docx]

|  |  | **Enhanced Algorithm Cases** | | |
| --- | --- | --- | --- | --- |
|  |  | **Yes** | **No** | **Total** |
| Chart Review | **Yes** | 27 | 1 | 28 |
|  | **No** | 14 | 6 | 20 |
|  | **Total** | 41 | 7 | 48 |
|  | | | | |
| Test Characteristics | **Sensitivity** | | 96.4 (27/28) | |
|  | **Specificity** | | 30.0 (6/20) | |
|  | **Negative predictive value** | | 85.7 (6/7) | |
|  | **Positive predictive value** | | 65.9 (27/41) | |
|  | **Youden’s index** | | 26.4 | |
|  | **F-score** | | 78.3 | |
